# Retraction: End-of-life discontinuation of destination therapy with cardiac and ventilatory support medical devices: physician-assisted death or allowing the patient to die?

**DOI:** 10.1186/1472-6939-11-20

**Published:** 2010-12-21

**Authors:** Mohamed Y Rady, Joseph L Verheijde

**Affiliations:** 1 Department of Critical Care Medicine, Mayo Clinic Hospital, Mayo Clinic, 5777 East Mayo Boulevard, Phoenix, Arizona, USA, 85054; Center for Biology and Society, School of Life Sciences, Arizona State University, 300 East University Drive, Tempe, Arizona, 85287, USA; 2 Department of Physical Medicine and Rehabilitation, Mayo Clinic Hospital, Mayo Clinic, 5777 East Mayo Boulevard, Phoenix, Arizona, USA, 85054; Department of Biomedical Ethics, College of Medicine, Mayo Clinic; and Center for Biology and Society, School of Life Sciences, Arizona State University, 300 East University Drive, Tempe, Arizona, 85287, USA

## 

The authors have voluntarily retracted this article [1] and it is no longer available for online public display because portions of the article are similar to a previous publication [2]. While there was no intention to use pre-existing work without appropriate attribution, the authors nonetheless extend their apologies to Dr. Miller and all others concerned.

## Pre-publication history

The pre-publication history for this paper can be accessed here:

http://www.biomedcentral.com/1472-6939/11/20/prepub

## References

[B1] Rady MY Verheijde JL End-of-life discontinuation of destination therapy with cardiac and ventilatory support medical devices: physician-assisted death or allowing the patient to die? BMC Medical Ethics 2010 11 15 20843327 10.1186/1472-6939-11-15 PMC2949779

[B2] Miller FG Truog RD Brock DW Moral Fictions and Medical Ethics Bioethics 2010 24 9 453 460 Published online: 7 July 2009 10.1111/j.1467-8519.2009.01738.x 19594726

